# Robust Automated SIFT-MS Quantitation of Volatile Compounds in Air Using a Multicomponent Gas Standard

**DOI:** 10.1021/jasms.3c00312

**Published:** 2023-11-21

**Authors:** Vaughan S. Langford, Kseniya Dryahina, Patrik Španěl

**Affiliations:** 1 Syft Technologies Limited, 68 Saint Asaph Street, Christchurch 8011, New Zealand; 2 J. Heyrovský Institute of Physical Chemistry, Czech Academy of Sciences, Prague 182 23, Czechia

**Keywords:** selected ion flow tube mass spectrometry, SIFT-MS, volatile organic compounds, VOCs, negative reagent ions, ion molecule reactions, nitrogen carrier gas, quantitation

## Abstract

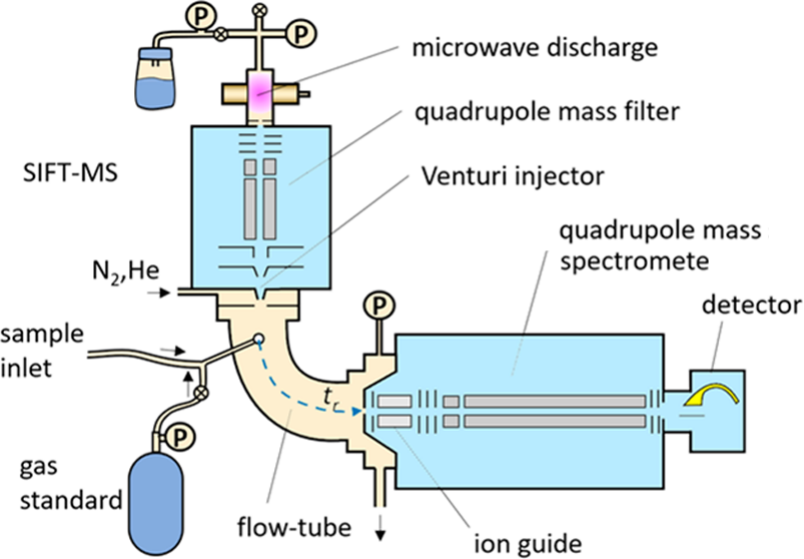

Selected ion flow tube mass spectrometry, SIFT-MS, has been widely used in industry and research since its introduction in the mid-1990s. Previously described quantitation methods have been advanced to include a gas standard for a more robust and repeatable analytical performance. The details of this approach to calculate the concentrations from ion–molecule reaction kinetics based on reaction times and instrument calibration functions determined from known concentrations in the standard mix are discussed. Important practical issues such as the overlap of product ions are outlined, and best-practice approaches are presented to enable them to be addressed during method development. This review provides a fundamental basis for a plethora of studies in broad application areas that are possible with SIFT-MS instruments.

## 1 Introduction

The selected ion flow tube, SIFT, is a well-established technique to study kinetics of gas phase ion–molecule reactions.^1^ Its first applications for online analyses of volatile compounds in ambient air, including very humid air such as exhaled breath, were described in 1996.^2−4^ Further research and development have resulted in the emergence of selected ion flow tube mass spectrometry, SIFT-MS, as a practical analytical technique.^5−7^ In principle, SIFT-MS applies gas-phase ion–molecule reactions^8^ to detect and quantify gases and vapors in air in real-time at trace concentrations (to subpart-per-billion by volume) because the standard reagent ions (H_3_O^+^, NO^+^, and O_2_^+•^; formerly termed “precursor ions”) do not react, or react only slowly, with the bulk constituents of air (N_2_, O_2_, H_2_O, Ar, and CO_2_).^6^ The resulting mass spectra thus reflect the composition of minor admixtures in the air sample based on the fundamental concept of mass spectrometry in general where each species of analyte results in one or more characteristic ion peaks on the spectra. SIFT-MS is used in a wide range of research, analytical and industrial and environmental applications.^9−12^

The original large laboratory-based SIFT-MS systems served as a basis for the development of the current small transportable instruments for real-time analyses of volatile compounds. Also, quantitation methods have been advanced to include a gas standard for more robust and repeatable analytical performance. In 2016, a dual-polarity ion source was introduced (adding O^–•^, OH^–^, O_2_^–•^, NO_2_^–^, and NO_3_^–^ reagent anions), opening a variety of volatile *inorganic* compounds to SIFT-MS analysis in addition to providing higher specificity for organics.^8,13^ However, while such methods are now widely adopted by industry, they are substantially different from the previously published methods lacking a gas standard.^14,15^ This article, therefore, serves to fill an important need not covered in recent review articles on the ion–molecule chemistry^8^ and applications^9^ of SIFT-MS. It does this in two main ways. First, the method of concentration calculation (in its ‘native’ units, ppbv) is explained in detail, including all inputs. Second, best-practice approaches to identifying the most sensitive and specific ions for analysis are provided, together with suggestions for deconvolution of overlaps where that occurs.

## 2 Ion-Molecule Reactions in SIFT-MS

Quantitation in SIFT-MS is based on the gas-phase chemical kinetics of ion–molecule reactions taking place over a well-defined reaction time. The primary focus of this section is to briefly introduce these reactions and define terminology that will be utilized in this article.

Reactions between ions and molecules in the gas phase underpin the SIFT-MS technique. They generally occur rapidly, providing highly sensitive probes of gas-phase composition in the ppbv range and below, without any requirement for sample preconcentration.^6,7^

Conventionally SIFT-MS has used three positively charged reagent ions (previously somewhat inaccurately called “precursor ions”) as standard: H_3_O^+^, NO^+^, and O_2_^+•^; so for simplicity, the examples below utilize only positively charged reagent ions though they equally apply to negative polarity. Equation 1 shows a two-body reaction illustrating how R^+^ reacts with the target compound (analyte) M to form a primary product ion P^+^ and neutral product(s) N.
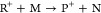
1where:

- R^+^ is the reagent ion,
- M is the target compound (analyte molecule),
- P^+^ is a primary product ion,
- N is the neutral product(s).

The rate coefficient, *k*, of this reaction determines the rate of change of the abundance of R^+^ as
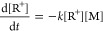
2The loss of R^+^ is balanced by the appearance of P^+^.

The common feature of all variants of mass spectrometry is that each analyte species is ionized to form ions with characteristic mass-to-charge ratios (*m*/*z*) and the resulting mass spectrum typically contains several ion peaks. This is the same in SIFT-MS and thus eq 1 is oversimplified for an ideal situation of a single type of product ion. There may, however, be multiple primary product ions due to different reaction channels. Let us consider a three-channel example, with product ions P_a_^+^, P_b_^+^, and MR^+^:
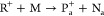
3a
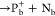
3b
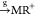
3cIn this example, reaction 3c is a three-body association channel forming an adduct ion; this is mediated by collisions with molecules of atoms of the carrier gas, g (usually He or N_2_), stabilizing the weakly bound adduct ions.

The relative rates of the reaction channels are described by branching ratios (*R*_b_) corresponding to fractions of 1. Note that association rate coefficient and branching ratios can be temperature, pressure and carrier gas dependent.^16^ The reaction branching in which chemically different product ions are formed must be distinguished from the situation where a single reaction channel occurs (i.e., eq 1), but product ions have several isotopologues. Consider, for example, chlorobenzene^17^ which reacts with NO^+^ and O_2_^+•^ via simple charge transfer to form C_6_H_5_Cl^+^. Since the natural abundance of Cl stable isotopes is 75.5% ^35^Cl to 24.5% ^37^Cl, corresponding product ions are observed in SIFT-MS mass spectra. Similarly, all organic molecules have ^13^C isotopologues that can represent substantial percentages of the ion signal. Proportions of various isotopologues should be called ion signal ratios. Note that, in terms of calculating concentrations, if only one primary product ion or isotopologue is utilized, then the branching ratio or ion signal ratio, respectively, is used to correct for the fact that all ions are not used.

In the SIFT-MS flow tube, primary product ions P^+^ may further react with a high-concentration species, X, to form a secondary product ion, S^+^ (eq 4).
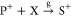
4In this example, the secondary ion product is formed in a three-body association reaction, again mediated by the carrier gas, g. Molecule X is most commonly water, H_2_O, for product ions in positive ion mode; in negative ion mode, CO_2_ also forms adducts effectively. Sometimes other secondary reactions can occur, especially for the product radical cations formed in the O_2_^+•^ reactions. Certain oxygen-containing VOCs (especially alcohols, aldehydes, ketones, and volatile fatty acids) also form adducts with their own product ions or with product ions from other minor analytes at relevant concentrations, starting typically around 1 part-per-million by volume (ppmv). To obtain the correct signal, and hence calculate the correct concentration for that channel, the secondary product ion signals must be measured and added into the primary product ion signal.

**Figure 1:**
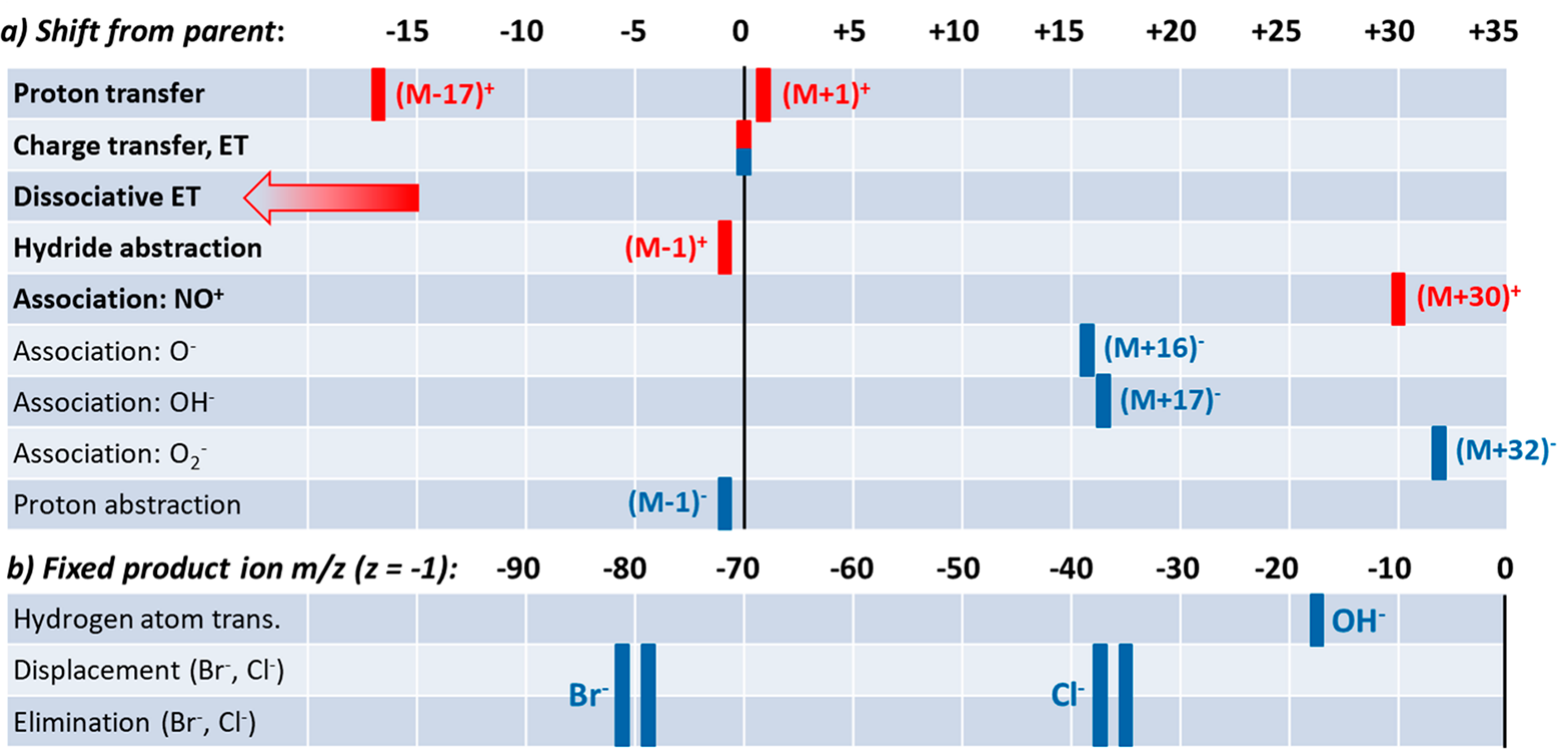
Visualization of (a) typical product ion shifts from the molecular ion for the most common reaction mechanisms and (b) common product ions formed in three negative ion mechanisms. Shifts shown in red and blue apply to positively and negatively charged reagent ions, respectively.

The reaction mechanisms of the positively charged reagent ions have been reviewed in detail previously.^6,7^ More recently, some discussion of the mechanisms of the negatively charged ions has been added, together with preliminary observations on the impacts of nitrogen carrier gas on ions of both polarities.^8,9^ It is unnecessary to cover the mechanistic details again here. The salient point, however, is that SIFT-MS reagent ions provide multiple reaction mechanisms to the user, which enable real-time, simultaneous analysis of chemically diverse volatile compounds (see, e.g., the fumigant application^9^), and selection of the most sensitive and specific product ions for the sample matrix. The relevance of a mechanism to a given compound depends on the specific gas-phase ionization energetics, and these generally follow trends for a given functional group. Figure 1 shows typical shifts or product ions formed for various mechanisms. Different combinations of mechanisms, leading to different *m*/*z* shifts, can frequently enable the method developer to resolve isobaric and even isomeric compounds using SIFT-MS. Worthy of note is the association reaction of NO^+^ with carbonyl containing species, such as carboxylic acids, esters, and ketones, which makes their SIFT-MS analyses more certain and robust.

## 3 Fundamentals of Quantitative Analysis Using SIFT-MS

The previous section briefly summarized ion–molecule reaction chemistry and how it is uniquely applied in SIFT-MS instruments. In this section, the conventional or “first principles” approach to quantitation in SIFT-MS is briefly summarized, before several approaches utilizing a gas standard are described in detail, with examples.

Quantitative analyses in SIFT-MS are based on calculations of concentrations of the targeted analyte compounds [M] in the flow tube reactor from the reagent, R^+^, and product, P^+^, ion signals based on the knowledge of the rate coefficient, *k*, and the branching ratios, *R*_b_, of reaction 3 between the selected reagent ion and the given analyte, and accurate determination of the reaction time. This concept is similar to the approach used in proton transfer reaction mass spectrometry, PTR-MS,^18^ and differs from the quantitation in most other MS techniques that is based on comparison with external or internal standards. SIFT-MS measurements of analyte concentration thus primarily rely on the correct assignment of product ions to the analytes and on the accurate determination of several physical parameters that relate the composition of sampled air to the composition of the low-pressure gas mixture in the flow tube. With these prerequisites, eq 2 can be used to calculate [M] from the rate of conversion of R^+^ to P^+^ when *k* is known, and the reaction time, *t*_r_, can be accurately determined. There are several key concepts and parameters on which SIFT-MS quantitation from first principles relies.

### 3.1 Reactivity and Transport of Ions

The reagent ions R^+^ are converted to product ions P^+^ in the flow tube by ion–molecule reactions with M (3). For most reactions, the rate of loss of R^+^ directly corresponds to the production rate of P^+^ (a notable exception being the associative detachment of negative ions, forming free electrons). Additionally, all ions can be lost by diffusion to the flow tube walls.^14,19^ The decrease of ion abundances during their residence time in the flow tube, *t*_*r*_, can then be expressed by solving the differential equation 2 with an additional term describing the diffusion loss as
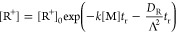
5where *D*_R_ is the diffusion coefficient for ions R^+^ in the carrier gas and Λ is the characteristic diffusion length related to the diameter of the flow tube. While it is possible to analytically solve the differential equation also for the formation and diffusion loss of P^+^, the solution can be simplified in the limit of the total product ion abundance  = 0
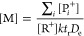
6In the flow tube, smaller ions tend to diffuse to the walls more readily than larger ions. This effect is described by the differential diffusion enhancement coefficient, *D*_e_, typically greater than 1, which depends on the difference of the diffusion coefficients of R^+^ and P^+^ and can be estimated from the geometrical size of the ions for the particular flow tube conditions.^20^

### 3.2 Rate Coefficients

The rate coefficients and branching of gas-phase ion–molecule reactions 3 can be experimentally determined by SIFT for each combination of R^+^ and M. Thousands of such reactions have been studied over several decades, and data on their kinetics provide an understanding of general trends relevant to SIFT-MS.^8,9,13^ A key principle is that at thermal energies the rate coefficients are limited by the collisional rate coefficient, *k*_c_, that theoretically describes the formation of the reaction intermediate ions due to the attractive force between the ion charge and the dipole of the molecule (both induced and permanent).^21^ It is well established that proton transfer reactions that are exothermic by more than 25 kJ/mol actually proceed with *k* = *k*_c_.^22^ Other types of reactions, such as charge transfer or hydride ion transfer under SIFT-MS conditions, can sometimes be slower: *k* ≤ *k*_c_. For nonpolar compounds, temperature invariant *k*_c_ can be calculated using Langevin theory^23^ from the molecular weights of the ion and the molecule and its polarizability only. For polar compounds, *k*_c_ increases with the dipole moment of the molecule *D* (typically up to two or three times) and reduces with temperature *T* (typically by 10 to 20% over the range accessible in the SIFT-MS instruments).

The data published on the kinetics of gas-phase reactions of the H_3_O^+^, NO^+^, and O_2_^+•^ ions with volatile analyte molecules cover thousands of reactions measured in He at ambient laboratory temperature (nominally 300 K). Much of these data can be reused with confidence after considering the following points:

- The rate coefficients for the polar analytes should be recalculated using the Su and Chesnavich^24^ parametrization for the temperature of the SIFT-MS instrument used for analyses. Nonpolar analytes are not affected.
- The rate coefficients for the association reactions (eq 3c) need to be experimentally determined under the conditions of instruments operating in N_2_ at elevated temperatures, as they will generally be unpredictably different from the 300 K He values.
- For the case of analyses that rely on a specific value of branching ratio, this value needs to be determined under the conditions of the actual instrument in use.

For practical use, the rate coefficients *k*, *k*_c_, and the reaction channel branching ratios can be collected in software-based libraries that facilitate the rapid development of quantification methods. A key principle to keep in mind is that SIFT-MS analysis is accurate only when the chemical composition of the analyte is correctly identified (for example, by gas chromatography MS) or known *a priori* (as is the case with industrial contamination).

### 3.3 Ion Signals, Their Measurement, and Relation to Ion Number Densities

To determine the ion abundances (in flow tube ion chemistry conventionally expressed as number densities in units cm^–3^), a fraction of flow tube gas containing ions is sampled into a high vacuum region of the downstream mass spectrometer. It can be reasonably assumed that the number of ions of each species transported by convection *via* a downstream sampling orifice will be proportional to their abundance in the flow tube gas. However, there can be differences in efficiency for each ion species, especially when an electric field is present that can cause ion drift in addition to convection flow. The ions sampled into the mass spectrometer are analyzed according to their mass-to-charge ratio (*m*/*z*) by the quadrupole mass spectrometer, in which the ion current transmission decreases with the *m*/*z* of the ion being selected.^25^ This is also influenced by the kinetic energy (velocity) of the ions in the quadrupole electrical field. Thus, the mass discrimination in the detection mass spectrometer must be accounted for.^19^ The detection efficiency of the electron multipliers decreases with *m*/*z*, and their linearity range is also limited. A reagent ion count rate that is too large results in nonlinearity of the ion detector (due to its dead time); this can be partially compensated for^14^ and can be in principle checked by the relative intensities of the ^18^O isotopologues of the reagent ions.^14^ Note that in the current generation of SIFT-MS instruments controlled attenuation of the reagent ion current is applied before the quadrupole mass spectrometer, as will be mentioned in Section 4.

Sufficient accuracy of the determination of reagent and product ion number densities can be achieved by a correct description of their transmission to the detector and characterization of its efficiency. The precision of this measurement is ultimately limited by the counting statistics; the standard error of the ion signal measurement corresponds to the estimated Poisson variance,^26^ a square root of the number of counted ions.^14^

Another important concept that needs to be understood when relating abundances of ionic species to ion signals is the presence of natural stable isotopes of elements present in the analyte molecules in addition to ^18^O (abundance 0.2%). The most important for SIFT-MS are ^13^C (1.1%) and ^34^S (4%). Each product ion species thus has several isotopologues that can represent a significant percentage of the ion signal (for example, protonated benzene C_6_H_7_^+^ will have the main peak at *m*/*z* 79 accompanied by over 6% signal at *m*/*z* 80). The fractions of the isotopologic signal should ideally be accounted for when calculating concentrations using eq 6.

### 3.4 Possible Sources of Overlaps

In real world analyses using SIFT-MS it is common that product ions from more than one analyte overlap at the same nominal *m*/*z*. The most obvious cases are isomers, for example, dimethylbenzene (xylene) and ethylbenzene (as will be discussed in detail in Section 5.3), for which the protonated molecules or radical cations cannot be distinguished by *m*/*z* (even high-resolution MS, such as time-of-flight, will not help). In some cases, the product ions are different, as is the case with aldehydes and ketones, where NO^+^ reacts by hydride ion transfer with aldehydes and by adduct forming association with ketones. It is thus a combination of data from several reagent ions that can help to identify isomers of analytes in analyzed air.

The above-mentioned isotopologues can also lead to overlaps. A good example is provided by acetone and acetic acid, which are present as volatile metabolites in human breath. The concentration of acetone can be one hundred times greater than that of acetic acid and the molecular weight of its ^18^O isotopologue overlaps with the main isotopologue of acetic acid. Similarly, trimethylamine overlaps with the ^13^C isotopologue of acetone. Such overlaps must be considered when analyzing multicomponent mixtures.

## 4 Advanced Implementation of SIFT-MS

Adoption of SIFT-MS instruments by industry demanded not only the miniaturization of large laboratory instruments that were originally used,^27^ but also enhanced usability. In research, users are typically technically competent, but not experts in ion chemistry and chemical kinetics, whereas in industry operators can be completely nontechnical. For technical users, appropriate software tools and instructional resources usually suffice, whereas for nontechnical users a completely “turnkey” solution for instrument operation is necessary. However, for both user scenarios, the concentration calculation should be the same. This section describes how concentrations are calculated through adaptation of the procedures of Section 3.

### 4.1 Arrangement of Advanced SIFT-MS Instruments

**Figure 2:**
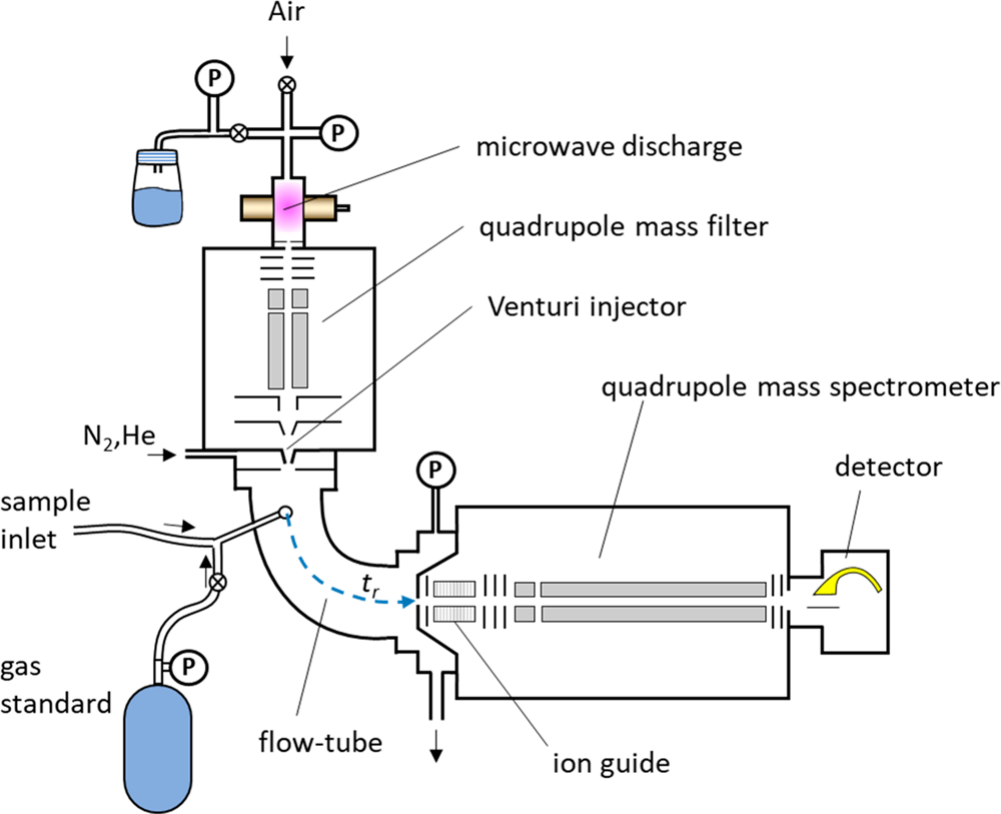
Schematic diagram of the arrangement of the advanced SIFT-MS instruments.

While the general principle of SIFT-MS has been described many times in scientific and commercial literature, it is important to outline the specific details of the implementation of this method in the state-of-the art instruments with a specific attention to the details relevant for quantification of the trace concentrations of volatile vapors in air. The general schematic of a typical advanced SIFT-MS instrument is shown in Figure 2 that covers the important sections (zones):

#### Ion Source

The positively charged reagent cations H_3_O^+^, NO^+^, and O_2_^+•^ and the reagent anions OH^–^ and O_2_^–^ are generated using a microwave discharge from a mixture of water vapor introduced from a bottle headspace and ambient air introduced via a filter. The remaining reagent anions O^–^, NO_2_^–^, and NO_3_^–^ are generated from ambient air only. The pressure in the source is approximately 0.7 mbar (0.5 Torr) for positive ions and 1.3 mbar (1 Torr) for negative ions. Effectively three ion source modes are thus available: wet positive, wet negative, and dry negative. For a given ion source mode, two or three species of reagent ions are generated simultaneously. To change from one set of ion source conditions to another (e.g., from positive ions to either of the two negative ion settings) takes up to 30 s. No compressed gases to generate the reagent ions are required. Depending on the desired polarity, appropriate voltages are applied to the ion source electrodes, and the mixture of ions generated is extracted to the quadrupole mass filter.

#### Selection Mass Filter

**Figure 3:**
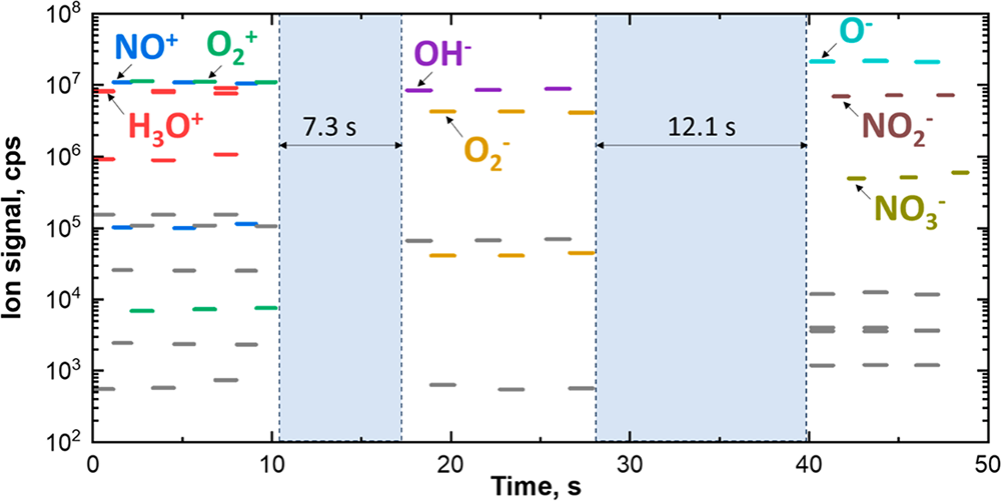
Three ion source modes for all eight reagent ions were utilized to collect selected ion monitoring (SIM) data over about 50 s. Blue shading indicates intervals for switching of the ion source to other operating conditions. Colored dashes show reagent ions and their hydrates, while gray dashes represent product ion signals (i.e., raw data) for acetaldehyde.

The quadrupole mass filter, together with the associated ion optics, is used to inject a beam of pure selected reagent ion species to the flow tube. Switching between ions of different *m*/*z* that are generated simultaneously in the discharge ion source can be achieved in milliseconds. Figure 3 shows a real example of the analysis of acetaldehyde using all eight reagent ions generated across the three ion source operating conditions. The ion injection energy is defined by the difference between the ion source discharge plasma potential and the potential of the Venturi injector used to introduce the filtered selected ions into the flow tube and must be sufficiently low to avoid fragmentation of the injected ions.^28^ The ion current collected by the Venturi injector electrode is measured in nanoamperes (nA) and can be used to obtain the injection mass spectrum.

#### Flow Tube Reactor

The reagent ions are injected into the carrier gas usually nitrogen (but helium, which was used exclusively in the past, remains an option) introduced via the Venturi injector, the flow rate of which is measured and stabilized by a mass flow controller (usually at a flow rate around 145 sccm, 2 Torr L/s). The reagent ions thermalize in multiple collisions with the molecules of the carrier gas and then move together with the flow of gas, into which a continuous flow of sampled air is added (usually at a flow rate of 22 sccm, 0.3 Torr L/s). The total pressure in the flow tube (*P*_g_) is continuously monitored and measured by an absolute membrane pressure gauge. The flow tube is heated by a closely fitting heater to typically 120 °C, the temperature is monitored and stabilized by accurate thermometric sensors. The total ion current reaching the downstream sampling electrode is continuously monitored by using an accurate pA meter. All of these parameters are recorded in the raw data files. A weak electric field is present at the entrance and exit of the flow-tube of the Voice200-series instruments to boost ion signals by accelerating and focusing the ion swarm and thus reducing the radial diffusion of reagent and product ions. This field is defined by potential differences among the Venturi injector, the flow tube, and the downstream sampling disk that are typically 25 V each.

#### Detection Mass Spectrometer

The ions are sampled via a pinhole orifice into an ion guide region. This ion guide was introduced in the Voice200 series, significantly improving the sensitivity and facilitating defined attenuation of the reagent ion signals. After focusing by an electrostatic lens, the ion beam enters a quadrupole mass spectrometer, where the analysis by *m*/*z* is performed across an available mass range of *m*/*z* 10–400. The *m*/*z* scale and peak widths are calibrated automatically during the performance check sequence by using the Syft Standard. Note that the peak positions and shapes are stable, allowing switching between nominal *m*/*z*, maximizing sensitivity for untargeted analyses. Targeted quantitation using SIFT-MS usually operates in the selected ion monitoring (SIM) mode of operation, recording only the *m*/*z* signals of interest.

Detection of signals from the quadrupole is achieved *via* a continuous dynode electron multiplier, operated in the pulse counting mode. A thresholding discriminator approach is used to determine ion count rates, and the resulting signals are presented as counts per second. Signal levels exceeding 10 million counts per second (cps) are typical for reagent ions on the latest commercial instruments. At these high count levels, dynamic attenuation is applied to ensure the ion rates are kept within the operating conditions of the detector (see below). Detector performance is checked by measuring the proportionality between reagent and product ions at a standard count level and at a detuned level. The counting system is assumed to be linear if the product/reagent cps ratios agree when measured at the standard and detuned levels for each product/reagent pair. The detuning is carried out automatically using the upstream axial bias to limit the reagent ions.

Conclusively, in combination with the real-time reagent ion switching in the selection mass filter (Figure 3), SIFT-MS instruments can acquire multiple product ion signals from independent reagent ions in SIM mode (see Sections 4.3 and 5.3 for discussions of concentration calculations from such data).

### 4.2 Inputs for Concentration Calculation

This subsection describes how several variables in the first-principles calculation (Section 3) are determined on instruments based on an integrated gas standard. Unmodified parameters are not mentioned.

#### The Instrument Performance Check

To ensure long-term stability, repeatability, and reproducibility, the current SIFT-MS instruments undergo a regular and automated performance check (formerly this multistep procedure was called a “validation”, but this is inappropriate usage). This check uses a certified gas standard containing several stable nonpolar gases at concentrations of about 2 ppmv with known concentrations of components to adjust the parameters involved in quantification. It is essential that the performance check is carried out under dry, leak-free conditions, especially when using nitrogen carrier gas. If the flow rate of the gas standard is identical to the sample flow rate, the instrument is effectively calibrated for the components of the standard mixture by defining the reaction time in the flow tube, *t*_r_ and the instrument calibration function, ICF, corresponding to the instrument sensitivity for different components of the gas standard. These parameters, together with the reagent ion attenuation and sample flow rate determination, are used directly in concentration calculation for all analytes available in the kinetics library, as described in brief here.

#### Reaction Time, *t*_r_

A low static voltage (typically 25 V) that is often applied between the Venturi injector and the flow tube, and between the flow tube and the sampling orifice, accelerates the reagent ions and thus shortens the reaction time below the value corresponding to carrier gas flow velocity. This means that the conventional approach to calculating the reaction time from carrier gas flow rate and pressure^14^ cannot be used. Instead, the ethene (C_2_H_4_) component of the certified standard is used to determine the reaction time during the instrument performance check sequence. The reaction of O_2_^+•^ with C_2_H_4_ results in a single product ion species P^*+*^ at *m*/*z* 28 (C_2_H_4_^+•^).^29^ If it is assumed that *m*/*z* 28 to 32 have the same ion transmission, then *t*_*r*_ can be calculated from the ratio of the product and reagent ion intensities as:
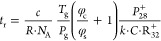
7Here:

- *c* is a number density of gas molecules (in cm^–3^) at 1 Torr pressure (3.894 × 10^15^),
- *R* is the gas constant (62.48 Torr K mol^–1^),
- *N*_A_ is Avogadro’s number (6.022 × 10^23^),
- *T*_g_ is the gas temperature in the flow tube (in Kelvin),
- *P*_g_ is the gas pressure in the flow tube (in Torr),
- φ_c_ is the carrier gas flow rate,
- φ_s_ is the sample flow rate in the same units,
- *k* is the rate coefficient for reaction of C_2_H_4_ with O_2_^+•^ (in cm^3^ s^–1^),
- *C* is the concentration of C_2_H_4_ in the gas standard (in ppbv),
- P_28_^+^ is the measured C_2_H_4_^+•^ (*m*/*z* 28) product ion signal (in cps),
- R_32_^+^ is the measured O_2_^+•^ (*m*/*z* 32) reagent ion signal (in cps).

The value of *t*_r_ is then stored in the instrument’s configuration files and listed in all subsequently obtained raw data files, as it can change during each instrument performance check sequence.

#### Ion Count Rates

Ion count rates are acquired during variable ion dwell time periods. The duration of these periods is limited by the software based on time and count limits. This protects the detector and prolongs its service life. In the raw data files, the values of the number of counts and the corresponding time period are recorded for each data point. The raw count rate is then calculated during data display and analysis as the ratio of these values:
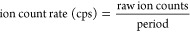
8A multiplier dead time correction is not required due to the application of reagent ion attenuation.

#### Reagent Ion Attenuation

Reagent ion count levels are typically in the 1–10 million count-per-second (Mcps) range in state-of-the-art SIFT-MS instruments and, as such, would be too large to be counted by the particle multiplier detector without using attenuation. Reagent ion signals are attenuated by using the ion guide bias voltage and are corrected by using a constant factor (usually factory determined). This correction is ordinarily invisible to users because it is applied to raw reagent ion signals prior to them being displayed on-screen or saved to file. Changes in the reagent ion attenuation factor may arise when instruments are retuned during maintenance or undergo repair.

#### Instrument Calibration Function, ICF

Mass discrimination and ion diffusion effects were traditionally treated separately as described previously.^6,19,25^ Accounting for these contributions on a compound-by-compound basis is challenging for most users of SIFT-MS instruments. Therefore, they have been combined into a single transmission parameter that is determined empirically as a function of *m*/*z* using the multicomponent gas standard. This standard contains known concentrations (nominally 2 ppmv) of seven nonpolar gases that cover the *m*/*z* range of the product ions up to *m*/*z* 236, do not undergo fragmentation, and do not form secondary product ions. Note that the ICF value is set to 1 by definition for *m*/*z* in the range from 28 to 32 and for *m*/*z* −32. The instrument calibration function value is determined for each component’s product ion *m*/*z* from the known *k* and previously determined *t*_r_ and stored in the instrument configuration files until the next instrument performance check sequence. This is described in detail below.

The ICF for positive polarity is set first. ICF values above *m*/*z* 32 are calculated using reactions of NO^+^ or O_2_^+•^ reagent ions with compounds at known concentrations in the standard:
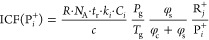
9Here:

- *k*_*i*_ is the rate coefficient for reaction of analyte *i* with reagent ion R_*j*_^+^ (in cm^3^ s^–1^),
- *C*_*i*_ is the concentration of analyte *i* in the gas standard (in ppbv),
- P_*i*_^+^ is the measured product ion signal for analyte *i* (in cps),
- R_*j*_^+^ is the measured NO^+^ (*m*/*z* 30) or O_2_^+•^ (*m*/*z* 32) reagent ion signal (in cps),
- ICF(P_*i*_^+^) is the value of the ICF for the product ion P_*i*_^+^ (dimensionless).

This approach applies up to *m*/*z* = 236 (octafluorotoluene). The ICF value at *m*/*z* 400 is linearly extrapolated from the values obtained at *m*/*z* 186 (hexafluorobenzene) and 236. The ICF value for *m*/*z* 19 is determined using compounds that react with both H_3_O^+^ and O_2_^+•^ (benzene and toluene) and for which the H_3_O^+^ product ion is offset by only 1 mass unit from the O_2_^+•^ product ion (78/79 and 92/93, respectively). The ICF value at the H_3_O^+^ product ion *m*/*z* (79 or 93) can be interpolated from the ICF value of the corresponding O_2_^+•^ product ion *m*/*z* (78 or 92) and the next highest measured ICF value (*m*/*z* 92 or 150, tetrafluorobenzene). The ICF value at the H_3_O^+^ product ion *m*/*z* can be accurately interpolated from the ICF value of the corresponding O_2_^+•^ product ion *m*/*z* and the next highest measured ICF value. Rearranging eq 9 to make the subject the ICF value for *m*/*z* 19 (ICF(R_19_^+^)), and including the nonunity ICF value for the product ion (ICF(P_*i*_^+^)), the formula used is:
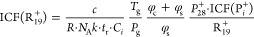
10

For negative polarity, the ICF values in the range *m*/*z* −28 to −400 are directly mirrored from the values at equivalent positive *m*/*z*. The ICF value at *m*/*z* −17 is evaluated using OH^–^ reactions with tetrafluorobenzene, hexafluorobenzene, and octafluorotoluene. The calculation is analogous to determination of the ICF for *m*/*z* 19. The ICF values for product ions of these OH^–^ reactions (at *m*/*z* −149, −183, and −235) are interpolated and then used to solve eq 10 for ICF(R_17_^–^). The three values obtained are averaged to give the value used in the ICF. The *m*/*z* −16 ICF value is determined from the reaction of O^–^ with hexafluorobenzene using the interpolated *m*/*z* −183 ICF value to solve for ICF(R_16_^–^).

The ICF is thus generated on completion of the instrument performance check sequence. An example of a normal ICF curve is provided in Figure S1 (Supporting Information). During data acquisition, these ICF values (stored in the configuration files) are linearly interpolated and extrapolated by instrument software to give the ICF for any product ion *m*/*z*. Note that this approach is similar to that utilized by PTR-MS.^30^ If a user makes a mistake, e.g., has another valve open to the flow tube additional to the Syft Standard calibration gas), the problem usually becomes evident through the generation of an ICF plot as a function of *m*/*z* that is of abnormal shape. Abnormalities can be evident as excessively high or low ICF values for *m*/*z* corresponding to H_3_O^+^, OH^–^ and O^–^, curvature at higher *m*/*z* that is too low or too high (noting that He carrier gas gives poorer high *m*/*z* transmission than N_2_), or a lack of smoothness in the curve. Unlike *Profile 3* instruments,^25^ the ICF does not take ion size and shape into account. Where discrepancies are significant and important, calibration of the compound can be carried out.

#### Sample Flow

Most advanced SIFT-MS instruments utilize a passivated, calibrated capillary for controlled introduction of sample^6^ from atmospheric pressure to the flow tube operating at *ca*. 1/1000 atm. For most applications, this is not explicitly user-entered into software for concentration calculation. Instead, a fixed flow rate (typically 22 sccm, 0.3 Torr L s^–1^) can be assumed, and the flow rate of the gas standard should be matched to the flow rate through the capillary. Clearly, good analytical practice requires that the calibration gas flow and the sample inlet flow (without the carrier gas flowing) match and are checked regularly. If the capillary flow has dropped significantly (typically by more than 20% from its original flow) then it should be cleaned or replaced.

### 4.3 Concentration Calculation Approach

The equation for determining the number density of the analyte in the flow tube for the simplest ion–molecule reaction (eq 1) is:
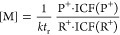
11Here:

- [M] is the number density of the analyte, M, in units of cm^–3^,
- R^+^ is the reagent ion count rate (in cps),
- P^+^ is the product ion count rate (in cps),
- ICF(P^+^) and ICF(R^+^) are the values of instrument calibration function for the ions P^+^ and R^+^ (dimensionless),
- *t*_r_ is the reaction time (in seconds); see eq 7.

When adduct formation occurs for the reagent ion R^+^ or secondary chemistry for the primary product ion P^+^, and accounting for the fact that multiple primary product ions may form, a more general equation is used to calculate [M]:
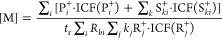
12Here:

- R_*j*_^+^ is the reagent ion signal (in cps) for the injected reagent ion (*j* = 0) and its hydrate ions (if appropriate; *j* = 1, 2, 3),
- *k*_*j*_ is the rate coefficient for reaction of reagent ion R_*j*_^+^ with the analyte (in cm^3^ s^–1^),
- P_*i*_^+^ is the primary product ion *i* signal (in cps),
- S_*ki*_^+^ is the secondary product ion *k* derived from primary product ion *i* signal (in cps),
- ICF(R_*j*_^+^), ICF(P_*i*_^+^), and ICF(S_*ki*_^+^) are the values of instrument calibration function for the ions given in parentheses (dimensionless),
- *R*_*bi*_ is the branching ratio for primary product ion *i* (0 < *R*_*bi*_ ≤ 1; i.e., converting from percentage to fraction).

Note that several approximations are included in this calculation. The contributions of the hydrated reagent ions R_*j*_^+^ are assumed to correspond to their signal at the downstream of the flow tube while in reality R_0_^+^ (e.g., H_3_O^+^) is converted to R_*j*_^+^ (e.g., H_3_O^+^(H_2_O)_*j*_) gradually along the flow tube^31^ and some of the hydrates formed may be fragmented on sampling. Also, the primary product branching ratio, *R*_b_, is taken as a single value (usually given in the library for H_3_O^+^ reaction only) irrespectively of the reagent ion’s hydration degree. The inaccuracy caused by this is usually negligible as long as the *k* for H_3_O^+^ is similar to that for H_3_O^+^H_2_O.

Instead of summation in the numerator of eq 10, current instrument software individually calculates the concentrations from each primary product ion P_*j*_^+^, its *R*_b_, and the associated secondary product ions selected for the analyte in the method. Thus, several [M]_*i*_ are calculated using:
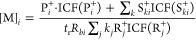
13

Next, the concentration in the sample itself is determined using dilution of sample flow into the carrier gas flow.

The conversion from [M]_*i*_ in the flow tube in the units of cm^–3^ to the corresponding concentration *C*_M*i*_ in sampled air expressed in parts-per-billion (ppbv) is achieved by considering the dilution of the sample flow in the carrier gas:
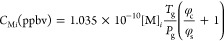
14These concentration values, obtained from *individual* primary product ions, are automatically saved point-by-point to the raw data files. Automated handling of these values is determined by the user in the method setup. The most-used reporting approach compares concentrations obtained with the individual ions and rejects those above a user-defined threshold (or “tolerance”; usually 20% higher than the lowest measured reading). All concentration measurements that lie ≤ 20% higher than the lowest reading are averaged, and this result is reported. This is a very powerful tool for automated interference rejection in turnkey SIFT-MS applications or for optimized methods in research. However, it is strongly recommended that when a method is being developed, or used to analyze a different matrix, then results for *individual* primary product ions should be inspected carefully, because missing secondary chemistry could mean that a false low reading will be calculated. See Section 5.

### 4.4 Example Concentration Calculations

Calculations of analyte concentrations for three typical samples are documented in detail in the Supporting Information.

Table S1 shows the full calculation of the chlorobenzene concentrations for the charge transfer product formed through the reaction with O_2_^+•^,^32^ both in terms of the individual ^35^Cl ^37^Cl isotopologues and the final concentration reading as it would be reported. These data are sourced from Perkins et al. 2021^33^ and correspond to the 100-ppb sample (in aqueous solution) analyzed as part of linearity validation. A Voice200*ultra* instrument operating on helium carrier gas was utilized here with automated headspace analysis, so there is a makeup gas correction at the conclusion of the calculations to account for sample dilution in the instrument inlet. This step is not required ordinarily but is included in all three examples.

Full calculation of ethanol concentrations determined using the H_3_O^+^ and NO^+^ primary product ions (each with a single secondary product ion—the water adduct) is shown in Table S2.^34^ These concentration data were extracted from a full-scan analysis of Parmesan cheese (one replicate of the Sainsbury’s “Taste the Difference” product in a recent application note^35^). These data were acquired on the same instrument and illustrate the handling of secondary chemistry and the tolerance procedure (Section 4.3) across different reagent ions.

The final example (Table S3) involves the reactions of hexanal with the H_3_O^+^ and NO^+^ reagent ions^36^ measured in the context of a study of paper odor^37^ (first replicate of sample 1). While the NO^+^ reaction is simple (i.e., yields a single product that does not have noticeable secondary chemistry), two primary product ions are formed with H_3_O^+^. The *m*/*z* 83 product ion is formed by rapid water elimination following protonation and has no secondary chemistry (according to the in-built library). However, the other product ion (*m*/*z* 101) arising from proton transfer does (119). This time, the data were acquired using the full scan mode on a Voice200*ultra* operating with nitrogen carrier gas (hence, the different flow tube pressure and carrier gas flow compared to the other examples), and concentration data were extracted subsequently using library kinetic data. The calculated data required user override of the automated tolerance function, because the H_3_O^+^ product ion at *m*/*z* 83 is significantly underreporting the hexanal concentration. The H_3_O^+^ reaction with hexanal needs to be re-examined in nitrogen carrier gas, because preliminary data suggest a significant reduction in branching ratio for *m*/*z* 83 (5%) compared to the original 1997 helium data,^36^ where the *R*_b_ for *m*/*z* 83 was reported as 50%, and (noting that the more recent helium carrier gas study gave 14 to 18% depending on humidity).^38^

### 4.5 Applying a Conventional Calibration Approach for Routine Laboratory Analysis

An emerging approach to quantitation involves applying SIFT-MS instruments as you would most conventional instruments^39^ by generating a calibration curve.^40^ Since this approach simply relies on correlation of measured signal to known concentration, the calculation does not rely on sample flow rate, diffusion, mass discrimination, reaction time, etc. In practical terms, this approach is easily accomplished on instruments that have an integrated autosampler. Standards can be prepared in an analogous manner to GC/MS, although careful attention must be given to nonaqueous solvent content.^39^

**Figure 4:**
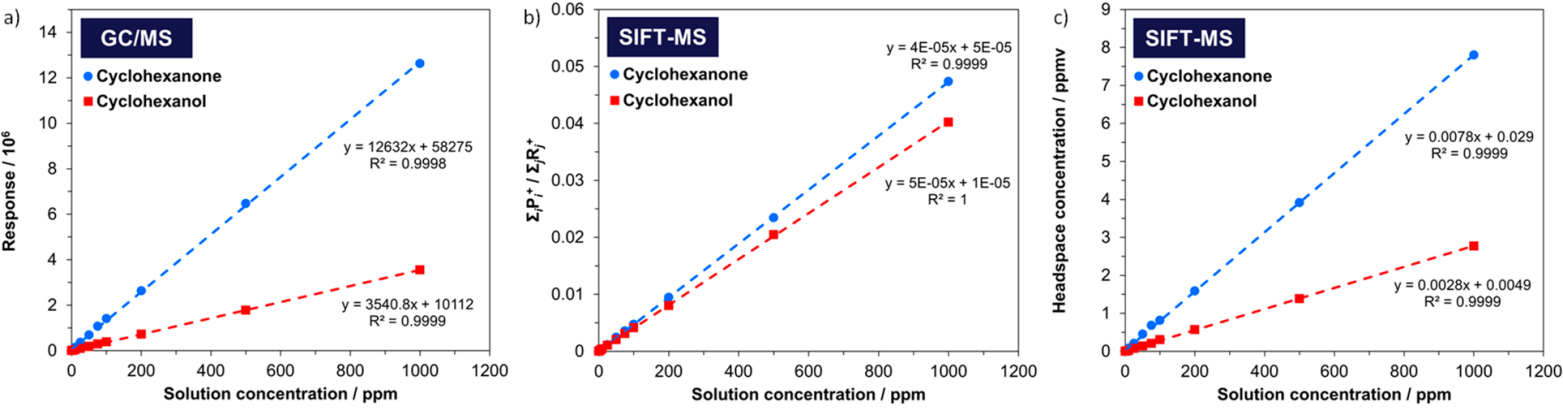
Calibration curves for headspace analysis using (a) GC/MS (raw response), and (b) normalized signal and (c) headspace concentration in ppmv for SIFT-MS. Adapted from Hastie et al.^41^

SIFT-MS calibrations tend to be valid for longer duration than is the case for GC/MS. The primary reasons for this are that (1) there is no retention time drift since the chromatographic column is eliminated in SIFT-MS, (2) the SIFT-MS ion source and detection system are physically separated from the flow tube, so they are much less prone to soiling, and (3) any drift in reagent ion signal intensity is mitigated by taking the ratio of the total product ion signal to the total reagent ion signal ([P^+^]/[R^+^]). In practical terms, the most convenient normalized measurements are the SIFT-MS headspace concentrations^17^ calculated in ppbv according to Section 4.3. Example calibration curves from a headspace study of cyclohexanone and cyclohexanol are shown in Figure 4.^41^

## 5 Method Development and Data Processing Approaches Supporting Quantitative Analysis

Having briefly traced the fundamentals of ion chemistry as they apply to SIFT-MS quantitation and then looked at various approaches to calculating concentrations on commercial instruments (with several examples), this final section outlines how reliable quantitation can be assured by good practice in method development. At the outset: understanding the matrix and selecting of the most suitable product ions are the main tasks. After gathering data: thoroughly inspecting them and refining the analytical method accordingly.

### 5.1 Matrix Considerations for SIFT-MS

SIFT-MS is a direct analysis technique, and hence, any reactive compound in the gas it samples (e.g., solvent or ethanol in alcoholic beverages) will contribute to consumption of reagent ion signal, which in turn can push the instrument outside its linear range. That is, the linearity of any given analyte is related to the total load of reactive compound in the instrument flow tube.

**Figure 5:**
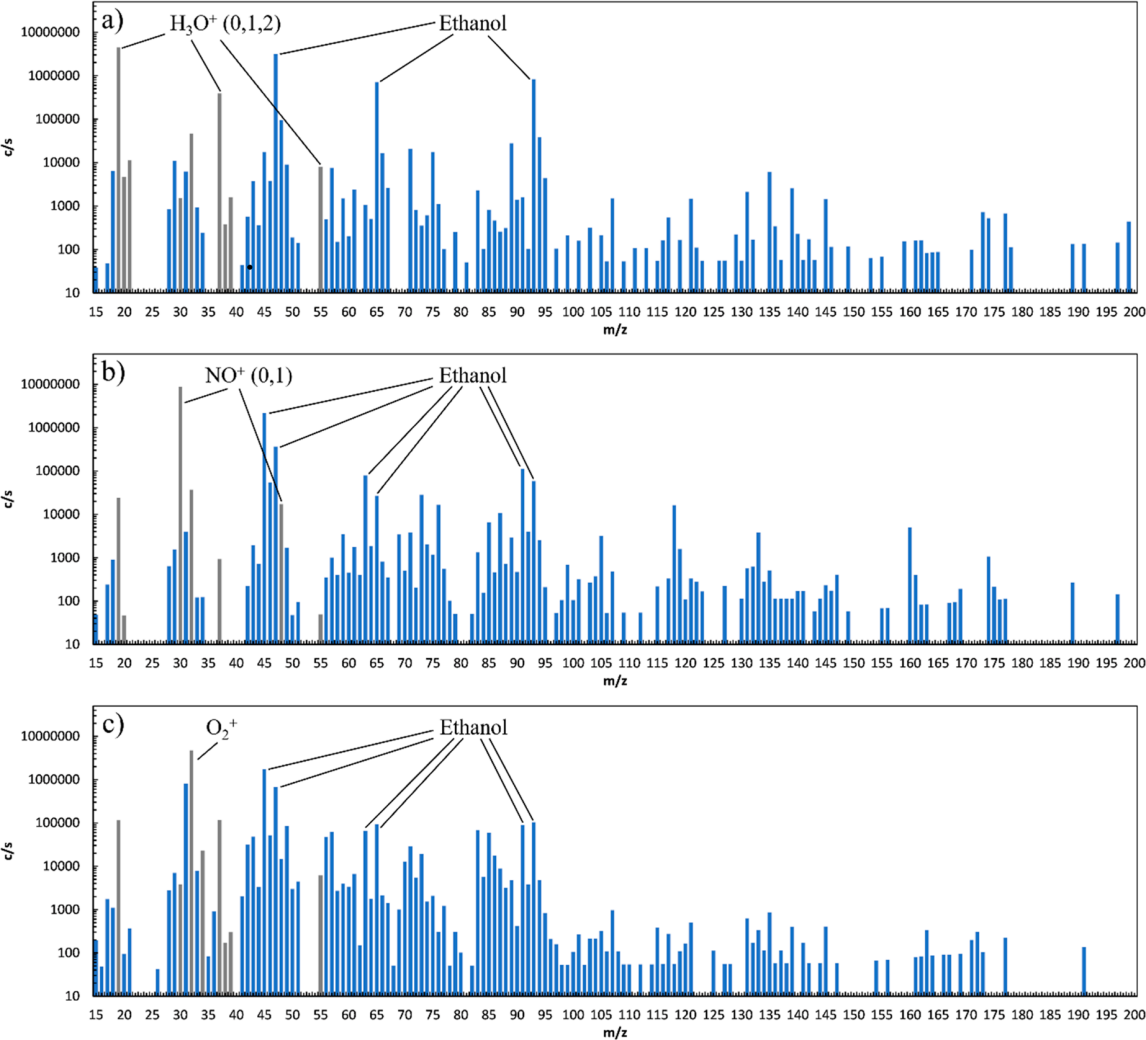
Representative SIFT-MS full scan spectra for (a) H_3_O^+^ reagent ions, (b) NO^+^ reagent ions, and (c) O_2_^+•^ reagent ions for the analysis of Stout beer (4.2% alcohol content), showing the features due to the ethanol matrix when diluted 10-fold in water. The reagent ions are shown in gray and the product ions in blue. Data from experiments.^35^

A good first step in the development of a new method is to run full scan analyses on a range of samples and compare these to the results obtained for zero air or laboratory air. The impact of the matrix on the reagent ion signals will be evident, and any major matrix volatiles identifiable. If signal levels indicate that the instrument is operating outside its linear range for some or all samples, then dilution of the samples (gaseous) or a reduction in the quantity of the solid- or liquid-phase sample used should be made. Even if no issues are observed due to the matrix, the full scan data are useful in determining the extent of the secondary ion chemistry—and hence the ions that should be included in the method to obtain correctly calculated concentrations. Figure 5 provides an example of the impact of the ethanol matrix in “Stout” beer, diluted 10-fold in water and analyzed using a SIFT-MS instrument with an integrated autosampler (a further 11-fold dilution of headspace in the instrument inlet).^35^ Note that the signal of protonated ethanol is almost as large as that of H_3_O^+^. Under these conditions SIFT-MS analysis should be conducted with caution, and matrix volatiles should be included in the SIM method, including any observed secondary chemistry, so that potential interference and quantitation impacts can be assessed immediately.^42^ Alternatively, a fast-GC separation stage^43^ or a thermal desorption unit^44^ could be added

### 5.2 Selection of Product Ions for Analysis

The most important considerations in the selection of target product ions for an analyte are usually specificity and sensitivity. Due to the real-time reagent ion switching in the SIFT-MS technique, typical SIM methods will acquire data for several primary product ions per analyte, where these exist, and apply automated interference rejection as described in Section 5.3.

Establishing that the analyte can be analyzed specifically is clearly the first step in SIM method development. If a product ion is specific to an analyte, then it will not be subject to interference. Use of method development and library software can assist in the identification of potential interferences. Note, however, that such software does not usually indicate whether low abundance isotopologues (e.g., ^13^C, ^18^O, and ^34^S) will interfere. Such issues sometimes arise in samples with adjacent analytes at very different concentrations.

Sensitive detection is usually assured when the reaction rate coefficient *k* is fast (typically 10^–9^ cm^3^ s^–1^ and above) and the branching ratio is close to 100%, although if reagent ion signals differ significantly, then this should be considered as well. In the above-mentioned software library, ions meeting these criteria are usually made default recommendations for analytical methods. However, exceptions occur where a product ion *m*/*z* falls on a reagent ion or its isotopologue, or at very common fragment ions (e.g., *m*/*z* 43 is very common with O_2_^+•^ due to C_3_H_7_^+^ and C_2_H_3_O^+^ fragment formation). For some compounds, high ion signal ratios do not occur due to significant fragmentation or the presence of several abundant stable isotopes (e.g., ^35^Cl and ^37^Cl, ^79^Br and ^81^Br, and in some circumstances ^32^S and ^34^S), resulting in reduced sensitivity per product ion. On the positive side, such isotopic signatures confirm the presence of the element in the analyte and, when not subject to interference, can be averaged manually to improve the signal-to-noise ratio.^17^

Generally, use of product ions with very small branching ratios and/or slow reaction rate coefficients (typically *R*_b_ < 10% and *k* < 10^–10^ cm^3^ s^–1^) are discouraged because calculation of the concentration using eq 14 will effectively amplify noise and/or any interference compared to more sensitive ions. For some analytes, however, one does not have another option due to limited available reactions with standard SIFT-MS reagent ions (e.g., methane).^45^

The method developer must also consider the impact of secondary chemistry on the selection of analyte product ions. As mentioned in Section 5.1, acquiring full scan data prior to SIM method development greatly assists evaluation of the secondary chemistry that is occurring and guides selection of product ions for data acquisition. Any secondary product ions that are likely to occur in samples must be included in the method; otherwise, the concentration will be under-reported for that primary product. Secondary product ions are mostly water adducts, but oxygenated VOCs – such as methanol, ethanol, acetaldehyde, acetic acid, and acetone – from the low parts per million by volume range upward can form dimers and mixed dimers. These may not be in the library in some cases – especially for mixed dimers – and they may interfere with other target compounds. (See Figure 5, where spectral features at *m*/*z* 91 for NO^+^ and O_2_^+•^, and at *m*/*z* 93 for all reagent ions arise from ethanol primary product ions interacting with excess free ethanol molecules in the flow tube.) Such issues can be mitigated through sample dilution even when the SIFT-MS instrument remains within its linear range because secondary product ion formation is strongly concentration dependent. Note, also, that measurement of secondary ions spreads the primary product ion intensity and costs more acquisition time; hence, sometimes a lower-sensitivity primary product ion with no secondary chemistry is a viable alternative, especially when time response is a key criterion of the method.

### 5.3 Dealing with Interference Issues

When a target compound is subject to interference and cannot be detected with sufficient sensitivity using unique primary product ions (if any), a deconvolution approach may or may not be feasible. Different scenarios are addressed here.

#### Subtraction Approaches

The subtraction of an interfering compound is possible where a total measurement can be made of the two compounds, and the interferent can be measured independently and with good signal-to-noise (to avoid propagation of measurement uncertainty). This approach works well for interferences by isotopologues and primary product ions. However, interference by secondary product ions is not easily correctable and can only be accomplished by either (1) analyzing samples at constant humidity or interferent concentration after first calibrating the interferent secondary chemistry under those conditions or (2) creating a concentration-dependent calibration of interferent secondary chemistry and utilizing this for prediction of the interference contribution. These corrections are outside the scope of this article.

##### Isotopologue Interference

This interference typically occurs when a ^13^C or ^18^O isotopologue of a more abundant compound interferes with a lower concentration compound at 1 or 2 *m*/*z* higher, respectively. A good example is afforded by the *N*,*N*-dimethylformamide (DMF; MW 73 g mol^–1^) interference with *N*-nitrosodimethylamine (NDMA; MW 74 g mol^–1^), where high-sensitivity detection of NDMA is important to the pharmaceutical industry.^46,47^ The ion–molecule reaction chemistry is summarized in Table 1 (NDMA;^48^ DMF^49^). NDMA has three primary product ions that provide high sensitivity, but each can be interfered with by ^13^C-DMF.

**Table 1:** SIFT-MS Reaction Chemistry (Rate Coefficient (*k*), Product Ion Formulae, Mass-to-Charge Ratio (*m/z*), and Branching Ratio (*R*_b_ as%)) for NDMA and DMF, a Potential Interferent^a^

	*N*-nitrosodimethylamine (NDMA)				*N*,*N*-dimethyformamide (DMF)			
reagent ion	*k*	formula	*m***/***z*	*R*_b_	*k*	formula	*m***/***z*	*R*_b_
H_3_O^+^	4.9	**(CH**_**3**_**)**_**2**_**N**_**2**_**OH**^**+**^	**75**	**100%**	3.3	**C**_**3**_**H**_**7**_**NOH**^**+**^	**74**	**95%**
						CHO^+^	29	5%
NO^+^	3.8	**(CH**_**3**_**)**_**2**_**N**_**2**_**O**^**+**^	**74**	**100%**	2.5	**C**_**3**_**H**_**7**_**NO**^**+**^	**73**	**45%**
						**C**_**3**_**H**_**6**_**NO**^**+**^	**72**	**45%**
						C_3_H_7_NONO^+^	103	10%
O_2_^+•^	4.1	**(CH**_**3**_**)**_**2**_**N**_**2**_**O**^**+**^	**74**	**95%**	2.9	**C**_**3**_**H**_**7**_**NO**^**+**^	**73**	**95%**
		(CH_3_)_2_N^+^	44	5%		C_3_H_7_^+^	43	5%

a Values in bold show the major product ions that are used for quantitation.

This interference is easily corrected by subtraction from the apparent NDMA signal using eq 15.
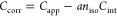
15Here:

- *C*_corr_ is the concentration of target analyte M corrected for isotopologue interference (in ppbv),
- *C*_app_ is the apparent concentration of target analyte M (i.e., uncorrected for isotopologue interference) in ppbv,
- *C*_int_ is the concentration of interfering compound measured using analyte M kinetic data at the predominant isotopic ion (e.g., 1 *m*/*z* less for ^13^C) in ppbv,
- *a* is the isotopic abundance of the element that is causing the interference,
- *n*_iso_ is the number of atoms of the element in the interfering compound.

##### Primary Ion Interference

Where one compound B interferes with compound A via a common product ion and only B can be analyzed independently, subtraction is achieved straightforwardly once the relative responses of A and B at the interfered *m*/*z* are accounted for. The general equation for subtraction is:
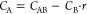
16Here:

- *C*_A_ is the calculated concentration of *C*_A_,
- *C*_AB_ is the apparent concentration of *C*_A_ for the product ion with which *C*_B_ interferes,
- *C*_B_ is the concentration of *C*_B_ measured independently of *C*_A_ (or any other interference),
- *r* is a response factor that accounts for the different sensitivities of *C*_A_ and *C*_B_; see eq 17 below.

The response factor *r* is determined by measuring a pure (diluted) sample of *C*_B_ at the interfered and unique *m*/*z*, where for the former the concentration is calculated by using *C*_A_ kinetic parameters. Hence, in the following eq 17, *C*_A*-*Pseudo_ is used, not *C*_A_.
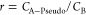
17This subtraction approach has been used successfully for many years in the turnkey fumigant detection application.^50^

Another example is analysis of ethylene oxide, a highly toxic fumigant and sterilant also used as a feedstock for polyethylene oxide polymers. It is readily detected using SIFT-MS (Table 2; ref (49)), but the most sensitive product ion (H_3_O^+^) is subject to regular interference by acetaldehyde.^36^ The ions of the O_2_^+•^ product fall at *m*/*z* common to many fragment ions or their isotopologues, which also renders them unhelpful in practice. In principle, ethylene oxide can be analyzed independently using its NO^+^ product ion at *m*/*z* 43, but because the reaction rate coefficient *k* is very slow, the sensitivity and hence limit of quantitation are degraded. Acetaldehyde can be analyzed independently via its *m*/*z* 43 product ion with NO^+^, and hence, subtraction can be achieved using the approach described above, while maintaining quantitation limits in the very low ppbv range. It is noted that very recent work^495^ using negative ions shows potential to obsolete the subtraction approach or at least provide the method developer with additional ions for evaluation in the sample matrices of interest (Table 2).

**Table 2:** Rate Coefficients for the Ion–Molecule Reactions of Interest for the SIFT-MS Analyses of Acetaldehyde and Ethylene Oxide^a^

	acetaldehyde (MW 44)					ethylene oxide (MW 44)				
reagent ion	*k*^c^	*k*^c^	formula	*m*/*z*	*R*_b_	*k*^b^	*k*^c^	formula	*m*/*z*	*R*_b_
H_3_O^+^	3.7	3.4	CH_3_CHOH^+^	45	100%	2.4	2.6	C_2_H_4_OH^+^	45	100%
NO^+^	0.6	0.6	CH_3_CO^+^	43	94%	0.1	0.1	C_2_H_3_O^+^	43	3%
			CH_3_CHO.NO^+^	74	6%			**C**_**2**_**H**_**4**_**ONO**^**+**^	**74**	**97%**
OH^–^	–	2.0	CH_2_CHO^–^	43	100%	–	0.1	C_2_HO^–^	41	5%
								C_2_H_4_OH^–^	45	5%
								C_2_H_3_O_2_^–^	59	90%
O^–•^	–	0.2	C_2_HO^–^	41	8%	–	0.07	C_2_HO^–^	41	96%
			C_2_H_3_O^–^	43	65%			C_2_H_3_O^–^	43	4%
			CO_2_H^–^	45	13%					
			C_2_H_3_O_2_^–^	59	14%					

a Branching ratios of the H_3_O^+^, NO^+^, OH^–^, and O^*–•*^ reactions with acetaldehyde and ethylene oxide. Values in bold show the major product ions.

b *k* (10^–9^ cm^3^ s^–1^) from previous data obtained at 300 K^49^

c *k* (10^–9^ cm^3^ s^–1^) from recent data obtained at 393 K.^495^

#### Linear Combination

When mutually interfering compounds have quite different branching ratios, but similar rate coefficients, an approach using a linear combination of product ion signals to calculate compound concentration^51,52^ has been helpful in resolving them. The prototypical example involves distinguishing ethylbenzene from the total xylenes based on the near-opposite branching ratios for the O_2_^+•^ reaction molecular ion (*m*/*z* 106) and fragment (*m*/*z* 91) product ion channels (Table 3).^32^ Reaction rate coefficients occur near the collisional rate for all ions, and the between-compound variability is less than 10% (i.e., within experimental uncertainty), so that for H_3_O^+^ and NO^+^ they provide a valid sum of all C_8_H_10_ isomers. Calibration curves are generated for the O_2_^+•^ product ions as a function of the relative proportions of ethylbenzene and a representative xylene isomer in a mix. The relative abundance of the two compounds can hence be determined in a given sample from these signals.^53^ The NO^+^ (or H_3_O^+^) reagent ion provides a measurement of the sum of the ethylbenzene and xylene isomers, to which this proportion can be applied to obtain the individual concentrations. Recently this approach has been applied successfully to drinking water,^17^ but because it requires that neither *m*/*z* 91 nor 106 product ions be subject to other interference, it is not viable in complex matrices such as wastewater. In that matrix, the total concentration of ethylbenzene and the xylenes was reported.^54^

**Table 3:** Detection Parameters for the Xylenes and Ethylbenzene Using the SIFT-MS Reagent Ions^32^^a^

	ethylbenzene (MW = 106)				xylenes (MW = 106)^b^			
reagent ion	*k*^c^	formula	*m*/*z*	*R*_b_	*k*^c^	formula	*m*/*z*	*R*_b_
H_3_O^+^	2.4	C_8_H_10_H^+^	107	100%	2.3	C_8_H_10_H^+^	107	100%
NO^+^	2.0	C_8_H_10_^+•^	106	100%	1.9	C_8_H_10_^+•^	106	100%
O_2_^+•^	2.0	C_7_H_7_^+^	91	70%	1.9	C_7_H_7_^+^	91	20%
		C_8_H_10_^+•^	106	30%		C_8_H_10_^+•^	106	80%

a Product formula (*m/z*); branching ratio as a percentage.

b The three xylene isomers (*o*-, *m*-, and *p*-) all react similarly with these reagent ions within 5% relative product abundance.

c *k* are given in the units of 10^–9^ cm^3^ s^–1^.

A hybrid approach, combining linear combination and subtraction, has been applied to speciating chloroform and dichloromethane,^33^ as well as chloroform and bromodichloromethane.^17^

#### Non-resolvable Interference

In some circumstances, it is not possible to distinguish between compounds at all. A prototypical example of this behavior is provided by the *o*-, *m*-, and *p*-xylene isomers, which have very similar ion–molecule reaction chemistries (Table 3).^32^ In this scenario, the sum of xylenes is reported. Furthermore, as noted above, if the matrix is more complex, then it may be that only a sum of ethylbenzene and the xylene isomers can be reported (using NO^+^ and/or H_3_O^+^ reagent ions).

When overlapped compounds exhibit significant dissimilarity in terms of reaction rate coefficients and branching ratio, then there will be greater uncertainty in the reported concentration, and this should be noted. See, for example, the reporting of total monoterpene isomers in Langford et al. 2020.^54^

### 5.4 Quality Assurance Data

This section briefly summarizes the basic checks that should be made to ensure that quality data are collected and reported.

#### Prior to Collecting Data

Follow the instrument manufacturer’s instructions for daily performance checks. These include (1) making visual confirmation that the ICF plot is of normal shape (see Supporting Information, Figure S1) and consistent with the previous automated performance check, (2) ensuring that the sample inlet is at its set temperature and the flow rate is normal, and (3) ensuring that reagent ion signal levels are normal and stable.

#### During Method Development

Ensure that sufficient matrix-matched blanks (especially in terms of humidity) are built into experimental planning—not just to check for carryover but also to provide reference signal levels to ensure that the instrument is not overloaded. Furthermore, for best results, ensure that method testing is conducted on as broad a range of samples as the method is anticipated to be used on.

In outline, the order in which data are checked is that (1) concentrations are being reported for analytes expected to be present, indicating that the sample is being delivered into the flow tube for analysis, (2) reagent ion signals are not consumed outside the linear range (usually, but not always, evident as the overreporting of concentrations), and (3) analyte concentrations are reporting in the expected range. The last point is significant and is effectively assessed by making thorough checks on the concentrations calculated for the individual primary product ions. Overreporting is usually due to interferences, whereas underreporting most frequently arises due to not handling secondary chemistry adequately in the method. A method can be optimized based on such findings.

#### Day-to-Day Data Acquisition Using a Well-Developed Method

Since any overload, carryover effects, and over- and under-reporting issues have been addressed in method development, outlier identification will be the main check in routine use. For example, significant loss of reported concentrations due to inlet blockage or a sample has abnormally high level of reactive VOCs, overwhelming the reagent ions and causing some carryover. When abnormal data are identified, more detailed checks of the sort listed for the “method development” phase should be made immediately, and the issue resolved.

Table 4 summarizes the recommended workflow for generating quality data using the SIFT-MS technique. As presented, the workflow presupposes that the principles of ion–molecule reaction chemistry and quantitation that are summarized in this article are understood, since these principles underpin reliable, quantitative SIFT-MS analysis.

**Table 4:** Recommended Workflow for SIFT-MS Analysis, from Conceptualization (i.e., Evaluating Whether SIFT-MS Is a Suitable Technique for the Application) Through to Day-to-Day Use of an Analytical Method

step	substep	recommended actions
**1. Suitability for SIFT-MS**	A. Matrix	Ensure that the:
		(1) matrix is not too complex for direct analysis to usefully analyze, and
		(2) total concentration of VOC is within dynamic range of SIFT-MS – if not, dilution will be required.
	B. Target compounds	(1) Confirm that target compounds are in the library.^a^
		(2) If a target compound is not in the library, do its ionization properties and do the volatility and/or partitioning suggest it will be detectable using SIFT-MS?
	C. Instrument configuration	Ensure that the available SIFT-MS instrument is appropriately configured: Carrier gas, sample inlet, sensitivity.
**2. Method development**	A. Full-scan analysis	Obtain representative samples that span the anticipated variation for this type.
		Analyze in full-scan mode to:
		(1) ensure that samples lie within the linear range,
		(2) ensure that target compounds are detectable, and
		(3) evaluate the extent of the secondary chemistry.
	B. Create targeted method	(1) Add compounds to method (from library), including major matrix volatiles.^a^
		(2) Ensure appropriate secondary product ions are included in the method (most commonly, but not necessarily only, water).
		(3) Assess probable interference issues and ensure that sufficiently sensitive ions (large *k* and *R*_*b*_) are available and selected.
	C. Sample analysis	Conduct regular instrument performance checks.
		Prepare suitable blanks/background checks.^b^
		Analyze blanks and test samples.
	D. Data analysis	Evaluate data in detail, ensuring that:
		(1) concentrations are reported for analytes expected to be present (no sampling or sample delivery issues),
		(2) reagent ion signals are within linear range (the instrument is not overloaded),
		(3) analyte concentrations are reporting in the expected range (individual primary product ions are reporting correctly).
		Ensure that blanks are not subject to carryover.
	E. Are data fit for purpose?	(1) Yes. Proceed to Step 3.
		(2) No. Minor issues, Step 2B. Major issues, Step 1.
**3. Method enhancements – optional**	A. More rigorous method testing and refinement	(1) ‘Stress’ the method to assess the impact of higher concentrations of matrix or target species.
		(2) Evaluate the performance of individual primary product ions for each analyte.
	B. Calibration	Establish calibration procedures if accuracy is a requirement (may not be required for all compounds in method).
	C. Method validation	If required, standard chromatography protocols are readily adapted to SIFT-MS.^c^
**4. Day-to-day method usage**	A. Instrument performance checks	Conduct all regular instrument performance checks, including sample flow rate.
	B. Sample analysis	Include blank/background measurements.
		Analyze samples according to method
	C. Data analysis	(1) Inspect samples for unusual results, e.g., very low/no reports (e.g., sampling failure or inlet blockage) or over-reporting (e.g., unusually high VOC).
		(2) When outlier measurements are encountered, follow Step 2D.

a If a target compound is not in the library, then it will need to be added.

b Good planning will enable multiple parameters to be assessed, including effectiveness of detection of compounds that are at low concentrations and carryover.

c See, for example Perkins et al.^33^

## 6 Conclusion

In targeted analyses using the advanced SIFT-MS instruments, calculations of concentrations occur automatically both onboard the instrument computer and in the desktop PC software application. This article documents for the first time the full details of the concentration calculations. This approach differs from that used on early SIFT-MS instruments, that are discussed in previous literature.^14,15^

Concentrations are calculated from the ratios of product ion signals to reagent ion signals using rate coefficients that are constant for a given reagent ion–analyte molecule pair, carrier gas type, pressure, and temperature. These do not change with the instrument settings or between the instruments. However, the reaction time and ion transmission (described by the instrument calibration function, ICF) are instrument-dependent and can vary temporally on a given instrument. These need to be calibrated on a regular basis, and this is achieved using the automated performance check conducted by the instrument software. Additionally, for valid quantitation, most instrument users need to ensure the integrity of sample flow in two ways. First, the inlet capillary is unobstructed. Second, the flow of the calibration standard is set to the same flow as the sample inlet capillary. By conducting these automated and manual checks regularly, reliable and reproducible quantitation of the analyte from the library is assured.

It should be noted, however, that the value reported for a targeted analyte in SIFT-MS is, strictly speaking, always an upper limit to the true concentration if method development has been conducted by using good practices. These include ensuring appropriate primary and secondary product ions are selected and that sample integrity is maintained in its delivery to the instrument. In this case, for example, if the measurement says there is 10 ppbv of benzene in sampled air, it is certain that the actual concentration of benzene is lower than or equal to 10 ppbv, even if there is unexpected interference. This is ideal for applications where the limits of VOCs must be assured.

Where interferences (or overlaps) with other ions occur, whether due to product ions or their isotopologues, the simultaneous use of multiple reagent ion–product ion pairs for a given analyte greatly minimizes the effect of such interferences. If a compound does not have a noninterfered product ion, various subtraction approaches have been utilized for effective quantitation.

Although this article has focused on quantitative analysis using commercial instruments, qualitative analysis using untargeted (i.e., full scan) SIFT-MS analysis combined with multivariate statistical analysis is gaining in popularity with a desire to apply in long-term studies.^55^ Example applications include rapid classification of Moroccan Argan oils,^56^ Mediterranean olive oils,^57^ breath,^58,59^ and recycled polymers.^12^ The present authors are not qualified to advise on statistical methodologies but would encourage those utilizing SIFT-MS as a “fingerprinting” tool (or VOC pattern analyzer) to ensure that input data of sufficient quality are acquired and used in the model. Of particular importance: (1) reagent ions and their ^18^O isotopologues should not be included, (2) samples should be well within the linear range (for matrix volatiles as well as target analytes), and (3) humidity should be kept constant. Failure to do so can result in the statistical method assigning importance to variables that are simply indicating humidity changes and/or high concentrations of volatiles that undergo secondary reactions; i.e., to spurious results.

In summary, careful method development, underpinned by a sound understanding of SIFT-MS ion–molecule chemistry and quantitation, should enable SIFT-MS users to obtain reliable quantitative data in a plethora of applications.

## Supporting Information Available

Figure S1, ICF Shape; Table S1, Example 1; Table S2, Example 2; Table S3: Example 3 (XLSX)

## Glossary of Symbols

R^+^: the reagent ion
M: the target compound (analyte molecule) M
P^+^: a primary product ion
N: neutral product(s)
*k*_c_: the collisional rate coefficient
*k*: the rate coefficient of the reaction (in cm^3^ s^–1^)
g: the carrier gas (usually He or N_2_)
*R*_b_: branching ratios
X: high-concentration species (H_2_O in positive ion mode; CO_2_ in negative ion mode)
Λ: the characteristic diffusion length related to the diameter of the flow tube
*D*_R_: the diffusion coefficient for ions R^+^ in the carrier gas
*D*_e_: the differential diffusion enhancement coefficient
*t*_r_: the ion residence time in the flow tube (reaction time)
*D*: the dipole moment of the molecule
*R*: the gas constant (62.48 Torr K mol^–1^)
*N*_A_: Avogadro’s number (6.022 × 10^23^)
*T*_g_: the gas temperature in the flow tube (in Kelvin)
*P*_g_: the gas pressure in the flow tube (in Torr)
φ_c_: the carrier gas flow rate
φ_s_: the sample flow rate
*C*: the concentration of C_2_H_4_ in the gas standard (in ppbv)
P_28_^+^: the measured C_2_H_4_^+•^ (*m*/*z* 28) product ion signal (in cps)
R_32_^+^: the measured O_2_^+•^ (*m*/*z* 32) reagent ion signal (in cps)
R_*j*_^+^: the reagent ion signal for the injected reagent ion (*j* = 0) and its hydrate ions (if appropriate; *j* = 1, 2, 3) (in cps)
P_*i*_^+^: the ion signal for primary product ion *i* (in cps)
S_*ki*_^+^: the ion signal for secondary product ion *k* derived from primary product ion *i* (in cps)
ICF(R_*j*_^+^),: ICF(P_*i*_^+^), and ICF(S_*ki*_^+^) the values of instrument calibration function for the ions given in parentheses (dimensionless)
[M]: the number density of the analyte M in the flow tube (in units of cm^–3^)
*C*_*Mi*_: concentration of the analyte M in sampled air (in ppbv)
*C*_corr_: the concentration of target analyte M corrected for isotopologue interference in ppbv
*C*_app_: the apparent concentration of target analyte M (i.e., uncorrected for isotopologue interference) in ppbv
*C*_int_: the concentration of interfering compound measured using analyte M kinetic data at the predominant isotopic ion (e.g., 1 *m*/*z* less for ^13^C) in ppbv
*a*: the isotopic abundance of the element that is causing the interference
*n*_iso_: the number of atoms of the element in the interfering compound.
*C*_A_: the calculated concentration of *C*_A_
*C*_AB_: the apparent concentration of *C*_A_ for the product ion with which *C*_B_ interferes
*C*_B_: the concentration of *C*_B_ measured independently of *C*_A_ (or any other interference)
*r*: response factor that accounts for the different sensitivities of *C*_A_ and *C*_B_
